# P53 and Parkin co-regulate mitophagy in bone marrow mesenchymal stem cells to promote the repair of early steroid-induced osteonecrosis of the femoral head

**DOI:** 10.1038/s41419-020-2238-1

**Published:** 2020-01-20

**Authors:** Fei Zhang, Wuxun Peng, Jian Zhang, Wentao Dong, Jianhua Wu, Tao Wang, Zhihong Xie

**Affiliations:** 1 Department of Orthopedics, The Affliated Hospital of Guizhou Medical University, Guiyang, Guizhou 550004 China; 2 Guizhou Medical University, Guiyang, Guizhou 550004 China

**Keywords:** Mitophagy, Apoptosis, Ageing, Mesenchymal stem cells, Diseases

## Abstract

Survival and stemness of bone marrow mesenchymal stem cells (BMSCs) in osteonecrotic areas are especially important in the treatment of early steroid-induced osteonecrosis of the femoral head (ONFH). We had previously used BMSCs to repair early steroid-induced ONFH, but the transplanted BMSCs underwent a great deal of stress-induced apoptosis and aging in the oxidative-stress (OS) microenvironment of the femoral-head necrotic area, which limited their efficacy. Our subsequent studies have shown that under OS, massive accumulation of damaged mitochondria in cells is an important factor leading to stress-induced apoptosis and senescence of BMSCs. The main reason for this accumulation is that OS leads to upregulation of protein 53 (P53), which inhibits mitochondrial translocation of Parkin and activation of Parkin’s E3 ubiquitin ligase, which decreases the level of mitophagy and leads to failure of cells to effectively remove damaged mitochondria. However, P53 downregulation can effectively reverse this process. Therefore, we upregulated Parkin and downregulated P53 in BMSCs. We found that this significantly enhanced mitophagy in BMSCs, decreased the accumulation of damaged mitochondria in cells, effectively resisted stress-induced BMSCs apoptosis and senescence, and improved the effect of BMSCs transplantation on early steroid-induced ONFH.

## Introduction

Effective treatment of early steroid-induced osteonecrosis of the femoral head (ONFH) is still a difficult and urgent problem in the field of orthopedics. Bone marrow mesenchymal stem cell (BMSCs) transplantation has been used to treat early steroid-induced ONFH^1–3^. Survival and stemness of BMSCs in the bone necrotic area are key to transplantation effectiveness^4–7^. After the femoral head suffers avascular necrosis, an oxidative-stress (OS) microenvironment forms in the osteonecrotic area^8–10^. The transplanted BMSCs undergo a great deal of stress-induced apoptosis and senescence in this microenvironment, which limits their efficacy^11,12^.

Central to OS is the release by impaired mitochondria of excessive reactive oxygen species (ROS) and apoptosis-inducing factors, which, in turn, increases telomere consumption, arrests the cell cycle, activates signaling pathways such as protein 53 (P53) and protein 38 mitogen-activated protein kinase (P38MAPK), and leads to cellular-stress-induced apoptosis and senescence^13–15^. Mitophagy is the most important way to eliminate damaged mitochondria from cells^16–18^, it not only promotes mitochondrial renewal but also reduces excessive ROS and apoptosis-inducing factors released by the damaged mitochondria^19^. Therefore, normal mitophagy is essential for maintaining cell redox homeostasis and promoting cell survival under OS conditions. However, under such conditions the level of mitophagy is always decreased, causing massive accumulation of damaged mitochondria and leading to stress-induced apoptosis and senescence in cells^20–22^.

At present, the known mechanisms of mitophagy include the phosphatase and tensin homolog (PTEN)-induced putative kinase protein 1 (PINK1)–Parkin, B-cell lymphoma 2 (Bcl2)/adenovirus E1B 19 kDa protein-interacting protein 3 (BNIP3)–Nix, and FUN14 domain-containing 1 (FUNDC1) pathways^19^. Studies have shown that under OS, mitophagy is mainly regulated by the PINK1–Parkin pathway^23^. When mitochondrial function is impaired, PINK1 aggregates in the extracorporeal membranes of damaged mitochondria and recruits Parkin there. Parkin is then phosphorylated to activate its E3 ubiquitin ligase activity and mediates ubiquitination of mitochondrial extracorporeal-membrane proteins, ultimately triggering mitophagy^24^.

It is well known that OS can lead to upregulation of P53^25–28^. In addition, recent studies have shown that P53 can bind to Parkin’s Really Interesting New Gene 0 (RING0) region, which interferes with Parkin’s biological function, affecting mitochondrial quality control, biosynthesis, kinetic regulation, and cellular redox homeostasis^19,29–31^. Parkin’s main function in mitochondrial quality control is to label damaged mitochondria via ubiquitin ligase activity, which, in turn, mediates mitophagy. Studies in mouse cardiomyocytes have shown that the binding of P53 to Parkin inhibits Parkin’s E3 ubiquitin ligase activity^29^. However, the significance of this process in BMSCs remains to be determined, and it is unclear whether it might interfere with mitophagy mediated by PINK1–Parkin. It is also not yet clear whether downregulating P53 expression while upregulating that of Parkin could significantly enhance mitophagy in order to resist stress-induced apoptosis and senescence of BMSCs, thereby improving the effectiveness of BMSCs transplantation.

In this study, we preliminarily explored the roles and mechanisms of P53 and Parkin in regulating mitophagy, as well as the effect of enhanced mitophagy on stress-induced apoptosis and senescence of BMSCs. We further evaluated the effect of P53–Parkin co-regulation of mitophagy on the repair of early steroid-induced ONFH by BMSCs transplantation.

## Results

### Repair of early steroid-induced ONFH in rabbits via BMSCs transplantation

**Fig. 1:**
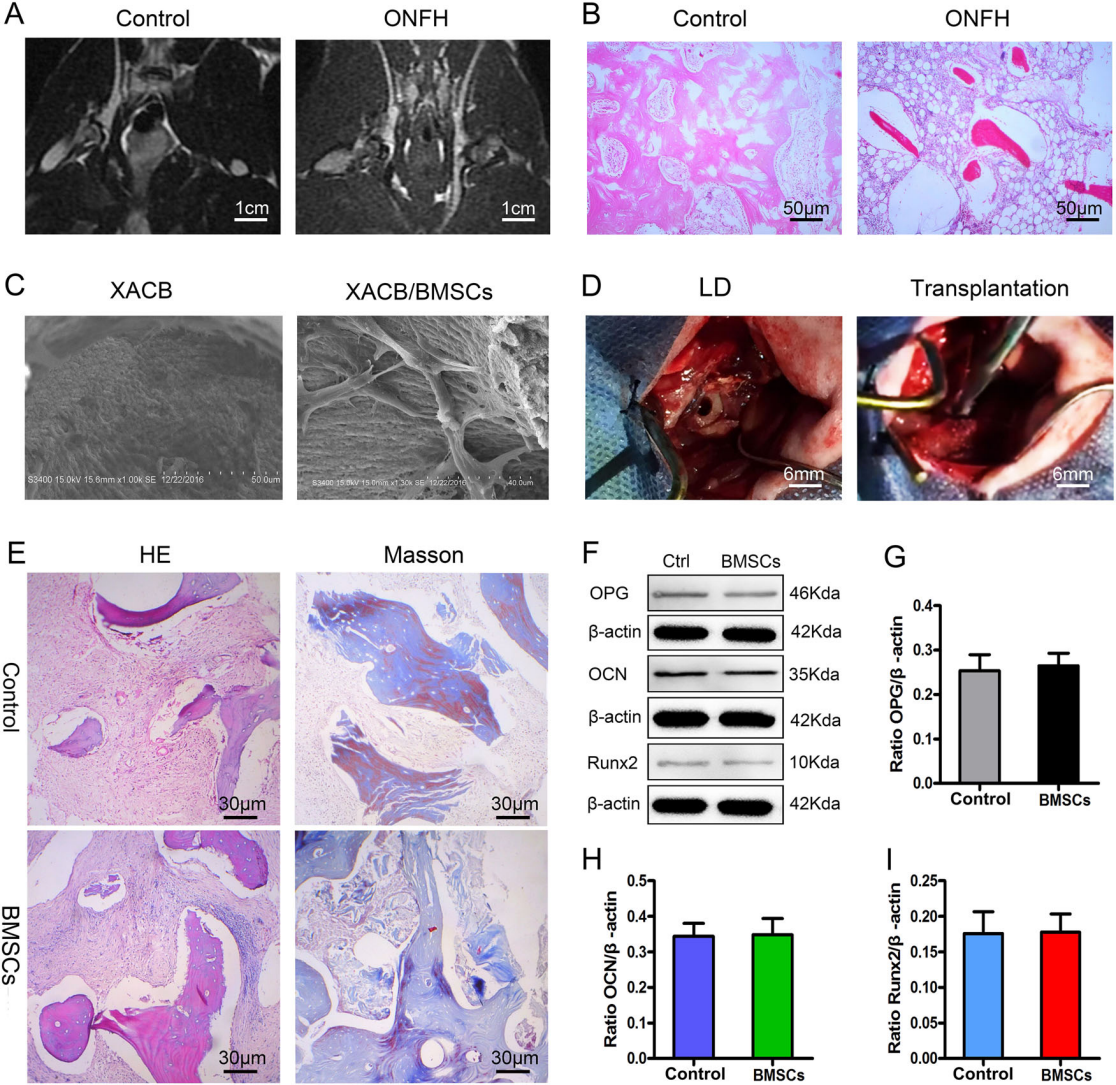
Osteogenesis of BMSCs in vivo. **a** Magnetic resonance imaging (MRI) examination of the ONFH model. **b** H&E staining of the ONFH model. Lipopolysaccharide (LPS) and methylprednisolone were used to build the model; saline was used as control. **c** Observation of XACB and XACB/BMSCs via scanning electron microscopy (SEM). **d** BMSCs repair of ONFH. Control was treated with lesion debridement (LD) without BMSCs implantation. **e** H&E and Masson staining to detect new-bone formation and maturation. **f**–**i** Western blot analysis of OPG, OCN, and Runx2 expression in osteonecrosis (*n* = 3). In (**g**–**i**), data are presented as means ± standard deviation (SD). Statistical significances were calculated by Student’s *t* test. BMSCs = bone marrow mesenchymal stem cells, ONFH = osteonecrosis of the femoral head, XACB = xenogeneic antigen-extracted cancellous bone, XACB/BMSCs = tissue-engineered bone, OPG = osteoprotegerin, OCN = osteocalcin, Runx2 = runt-related transcription factor 2.

BMSCs have strong self-renewal ability and multi-directional differentiation potential^32–34^. In order to study their osteogenic potential, we induced osteogenic differentiation of BMSCs. After 2 weeks, a large number of calcium nodules were formed, alizarin red and alkaline phosphatase (ALP) staining results were positive, and we confirmed that the BMSCs had strong osteogenic potential in vitro (Fig. S1). To observe BMSCs osteogenesis in vivo, we used lipopolysaccharide (LPS) combined with methylprednisolone to establish an early steroid-induced ONFH model in rabbits. At week 8, magnetic resonance imaging (MRI; T2-weighted image [WI]) showed mixed signals of different heights in the femoral head (Fig. 1a). Hematoxylin and eosin (H&E) staining showed that the medullary cavity was filled with a large amount of adipose tissue, the trabecular bone had become thinner, and no osteoblast-related cells were present (Fig. 1b). These results confirmed the success of our early steroid-induced ONFH model.

Subsequently, we inoculated BMSCs into xenogeneic antigen-extracted cancellous bone (XACB) for co-culture to create tissue-engineered bone (BMSCs/XACB). On day 6, we observed BMSCs growing on the surface of the XACB using a scanning electron microscope (SEM); these BMSCs had good biocompatibility (Fig. 1c). Finally, we transplanted the BMSCs/XACB into the rabbits to repair early steroid-induced ONFH (Fig. 1d). At 12 weeks postsurgery, we evaluated the repair of the femoral-head necrotic area by H&E staining, Masson staining, and detection of osteogenic markers such as osteoprotegerin (OPG), osteocalcin (OCN), and runt-related transcription factor 2 (Runx2). The results showed that only a small amount of new-bone tissue had formed in the necrotic area of the XACB group and the BMSCs/XACB group, and no mature bone tissue was found (Fig. 1e). Also, there were no significant difference in the area of new-bone formation and in levels of osteogenic markers (Fig. 1f–i). In conclusion, after tissue-engineered bone implantation into the femoral-head necrotic area, there was no significant difference in osteogenesis between the BMSCs/XACB and XACB groups. Therefore, we had reason to speculate that there might be important factors affecting the survival and osteogenic differentiation of BMSCs in necrosis of the femoral head.

### Excessive accumulation of damaged mitochondria led to stress-induced apoptosis and senescence of BMSCs

**Fig. 2:**
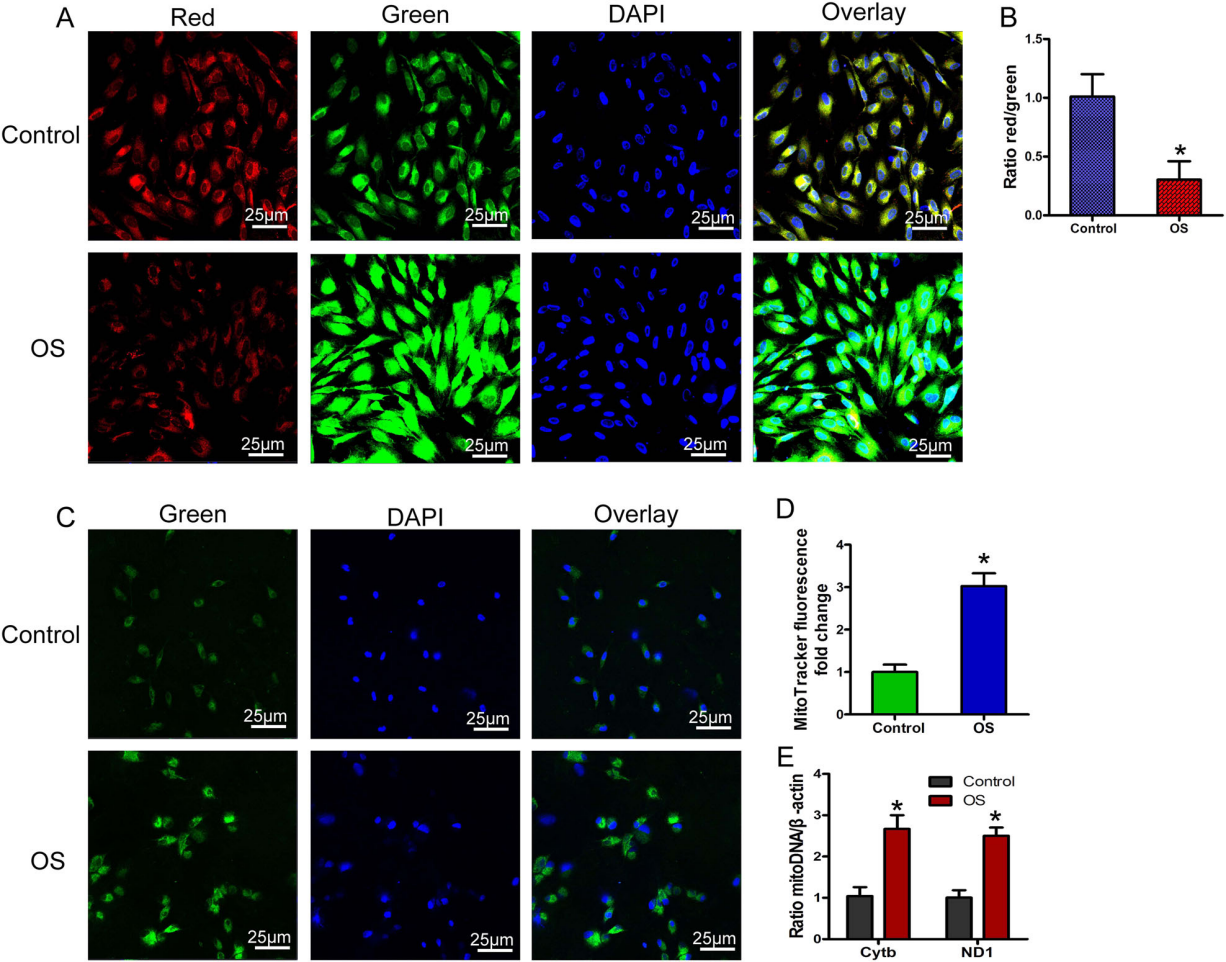
OS led to accumulation of damaged mitochondria. **a**, **b** Mitochondrial-membrane potential (MMP) detected by JC-1 (*n* = 4). **c**, **d** MitoTracker Green analysis of mitochondrial content (*n* = 4). **e** Quantitative polymerase chain reaction (qPCR) analysis of mitochondrial deoxyribonucleic acid (mtDNA; *n* = 4). In (**b**), (**d**), and (**e**), data are presented as means ± SD. Statistical significances were calculated by Student’s *t* test. Data were compared with the control group: *P < 0.05. H_2_O_2_ was used to simulate OS; L-DMEM was used as control. OS = oxidative stress; DAPI = 4′,6-diamidino-2-phenylindole; Cytb: cytochrome b; ND1 = mitochondrially encoded 1,4-dihydronicotinamide adenine dinucleotide (NADH):ubiquinone oxidoreductase core subunit 1.

In the avascular necrotic area of the femoral head, the function of the oxygenated mitochondrial respiratory chain is impaired by ischemia and hypoxia, producing excessive ROS. Moreover, after ischemic necrosis of the femoral head, inflammatory cells infiltrate, and the released inflammatory mediators also mediate ROS production by the inflammatory cells. Therefore, after necrosis of the femoral head, a local OS microenvironment forms^8–11^. In order to further study the effects of OS on BMSCs and then to treat the BMSCs, we used high-concentration H_2_O_2_ (1000 μM) to simulate an OS microenvironment^35^. We used JC-1 to measure mitochondrial-membrane potential (MMP), and MitoTracker Green staining and a quantitative mitochondrial deoxyribonucleic acid (mtDNA) assay to assess intracellular mitochondrial content. The results showed that after BMSCs were subjected to OS, MMP decreased, mitochondrial function was impaired (Fig. 2a, b), and the damaged-mitochondria content increased significantly in cells (Fig. 2c–e).

**Fig. 3:**
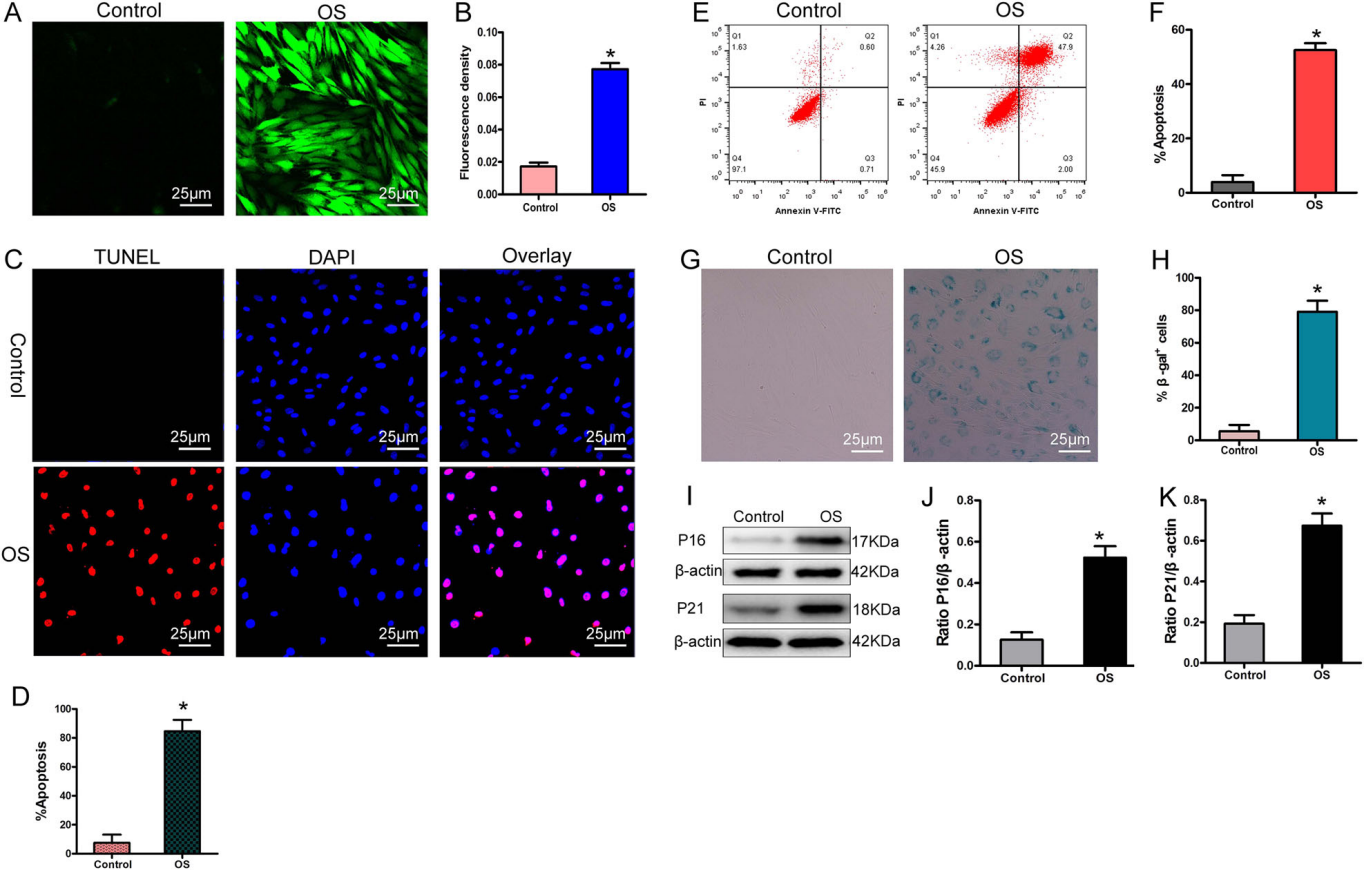
Accumulation of damaged mitochondria led to stress-induced apoptosis and senescence. **a**, **b** 2′,7′-Dichlorofluorescin diacetate (DCFH-DA) detection of reactive oxygen species (ROS; *n* = 4). **c**, **d** TUNEL–DAPI detection of deoxyribonucleic acid (DNA) damage and apoptosis (*n* = 4). **e**, **f** Annexin V–FITC and propidium iodide (PI) detection of apoptosis (*n* = 4). **g**, **h** Detection of β-gal activity by β-gal staining (*n* = 4). **i**–**k** Western blot analysis of P16 and P21 expression (*n* = 3). In (**b**), (**d**), (**f**), (**h**), and (**j**–**k**), data are presented as means ± SD. Statistical significances were calculated by Student’s *t* test. Data were compared with the control group: *P < 0.05. H_2_O_2_ was used to simulate OS; L-DMEM was used as control. TUNEL = terminal deoxynucleotidyl transferase deoxyuridine-5′-triphosphate nick end labeling; DAPI = 4′,6-diamidino-2-phenylindole; FITC = fluorescein isothiocyanate; P16 = protein 16; P21 = protein 21; β-gal = β-galactosidase.

Subsequently, we used a 2′,7′-dichlorodihydrofluorescein diacetate (DCFH-DA) fluorescent probe to detect ROS levels, terminal deoxynucleotidyl transferase deoxyuridine-5′-triphosphate nick end labeling (TUNEL) staining to detect DNA damage, Annexin V–FITC and propidium iodide (PI) staining to detect apoptosis, and β-galactosidase (β-gal) staining and examination of protein 21 (P21) and other senescence-associated protein levels to assess cell senescence. The results showed that with the increase of intracellular damaged-mitochondria content, intracellular ROS levels also increased significantly (Fig. 3a, b), and DNA damage increased as well (Fig. 3c, d). Eventually, a large number of BMSCs were induced to undergo apoptosis (Fig. 3e, f), β-gal activity increased in the surviving BMSCs (Fig. 3g, h), the expression of aging-related proteins such as P21 and protein 16 (P16) was also significantly increased (Fig. 3i–k), and the surviving BMSCs showed stress-induced aging. Therefore, our results showed that stress-induced apoptosis and aging of BMSCs were not only related to mitochondrial dysfunction but also related to accumulation of damaged mitochondria in cells.

### Enhancing mitophagy against BMSCs stress-induced apoptosis and senescence

**Fig. 4:**
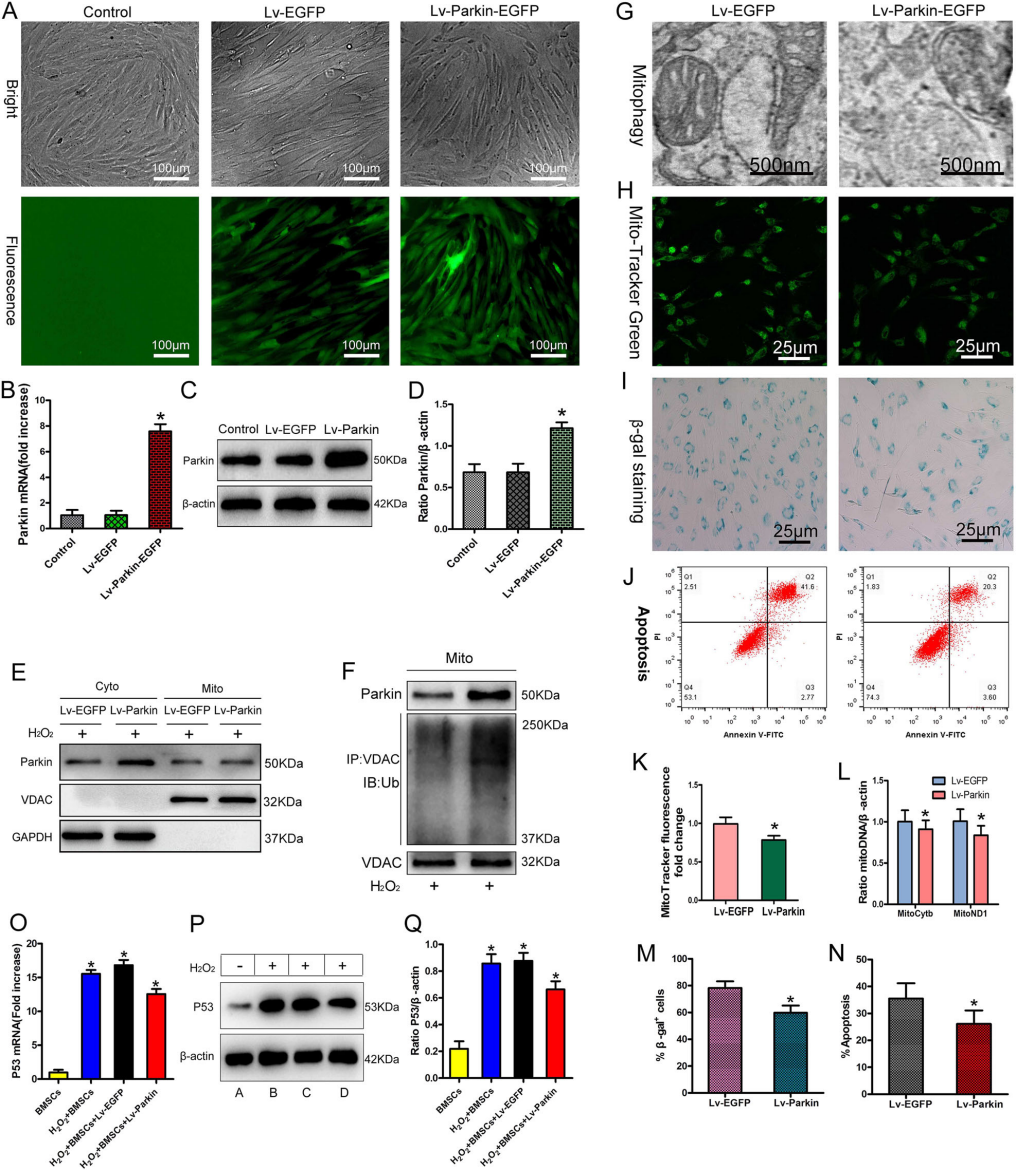
Enhancing mitophagy against stress-induced apoptosis and senescence in BMSCs. **a** Observation of fluorescent protein by inverted fluorescence microscopy (*n* = 4). **b** qPCR analysis of Parkin mRNA expression (*n* = 4). **c**, **d** Western blot analysis of Parkin expression (*n* = 4). Non-transfected BMSCs as control. **e** Western blot analysis of Parkin mitochondrial translocation (*n* = 3). **f** Immunocoprecipitation (IP) and immunoblot (IB) analysis of VDAC1 ubiquitination level (*n* = 3). **g** Observation of mitophagy by transmission electron microscopy (TEM; *n* = 4). **h**, **k** MitoTracker Green analysis of mitochondrial content (*n* = 4). **i**, **m** Detection of β-gal activity by β-gal staining (*n* = 4). **j**, **n** Annexin V–FITC and PI detection of apoptosis (*n* = 4). **l** qPCR analysis of mitochondrial deoxyribonucleic acid (mtDNA; *n* = 4). **o** qPCR analysis of P53 mRNA expression (*n* = 4). **p**, **q** Western blot analysis of P53 expression (*n* = 4). In (**p**), A = BMSCs, B = H_2_O_2_ + BMSCs, C = H_2_O_2_ + BMSCs + Lv-EGFP, D = H_2_O_2_ + BMSCs + Lv-Parkin. In (**b**), (**d**), (**k**–**o**), and (**q**), data are presented as means ± SD. Statistical significances were calculated by ANOVA and Student’s *t* test. In (**b**) and (**d**), data were compared with the control and Lv-EGFP groups separately; vs. control and Lv-EGFP: *P < 0.05. In (**k**–**n**), data were compared with the Lv-EGFP group: *P < 0.05. In (**o**) and (**q**), data were compared with the BMSCs group: *P < 0.05. Lv-EGFP = lentiviral vector-encoded green fluorescent protein; Lv-Parkin-EGFP = lentiviral vector-encoded Parkin-enhanced green fluorescent protein; mRNA = messenger ribonucleic acid; Ub = ubiquitin; VDAC1 = voltage-dependent anion-selective channel 1; P53 = protein 53; GAPDH = glyceraldehyde 3-phosphate dehydrogenase.

Stress-induced apoptosis and senescence in BMSCs are related to the accumulation of damaged mitochondria in cells^20–22,36^. Could enhancing mitophagy to clear these accumulated mitochondria effectively resist BMSCs stress-induced apoptosis and senescence? Mitophagy mediated by the PINK1–Parkin pathway is the most important way to clear damaged mitochondria^37,38^; mitochondrial translocation of Parkin is very important to mitophagy^39^, and therefore increasing the content of Parkin in cells can be expected to increase mitochondrial translocation of Parkin and thus enhance mitophagy. Therefore, we used lentiviral vector-encoded Parkin-enhanced green fluorescent protein (Lv-Parkin-EGFP) to transfect BMSCs. The results of quantitative polymerase chain reaction (qPCR) and western blot confirmed that Parkin was successfully overexpressed in BMSCs (Fig. 4a–d). We then used high-concentration H_2_O_2_ (1000 μM) to simulate OS in order to treat BMSCs for 24 h^35^, hoping that we could enhance mitophagy by interfering with the PINK1–Parkin pathway. However, our immunocoprecipitation and western blot results showed that under OS, mitochondrial translocation of Parkin and the ubiquitination level of voltage-dependent anion-selective channel 1 (VDAC1) were increased slightly after lentivirus-mediated overexpression of Parkin (Fig. 4e–f). Electron microscopy showed that the number of mitophagic bodies in the BMSCs also increased slightly (Fig. 4g), but levels of damaged mitochondria (Fig. 4h, k–l), apoptosis, and β-gal activity in Parkin-overexpressing BMSCs were not significantly decreased compared with the control group (Fig. 4i–j, m–n). In conclusion, upregulation of Parkin expression alone had limited effects on enhancing mitophagy and resisting stress-induced apoptosis and aging in BMSCs. However, it is interesting to note that expression of P53 in cells was significantly upregulated after OS was induced in each group of BMSCs (Fig. 4o–q). Recent studies have shown that P53 can bind to Parkin and interfere with its physiological functions^29–31^. This may interfere with Parkin-mediated mitophagy, limiting the ability of Parkin upregulation alone to enhance mitophagy.

**Fig. 5:**
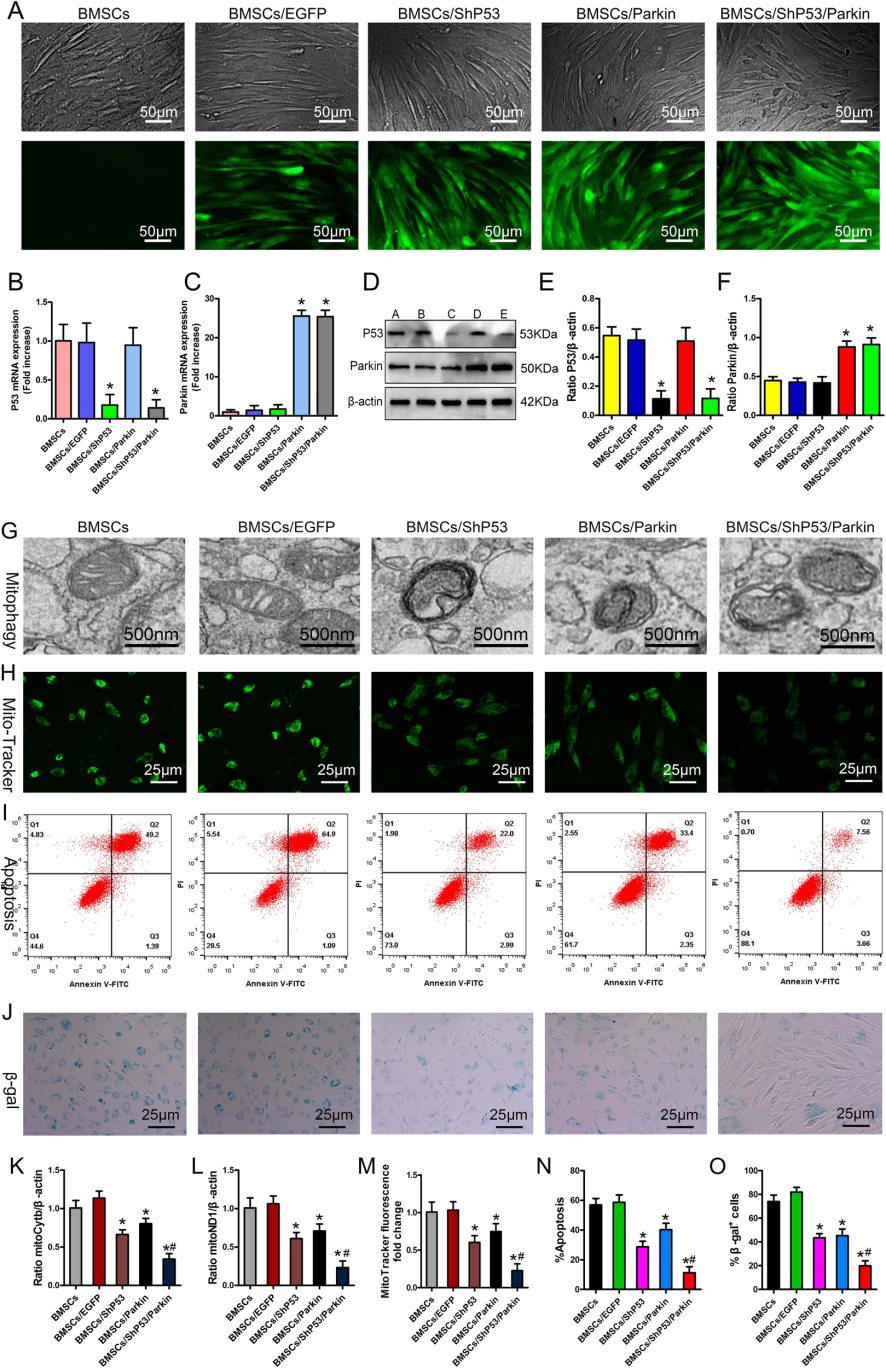
Effects of mitophagy co-regulated by P53 and Parkin on stress-induced apoptosis and aging. **a** Observation of fluorescent protein by inverted fluorescence microscopy (*n* = 4). **b**, **c** qPCR analysis of P53 and Parkin mRNA expression (*n* = 4). **d**–**f** Western blot analysis of P53 and Parkin expression (*n* = 4). **g** Observation of mitophagy by TEM (*n* = 3). **h**, **m** MitoTracker Green analysis of mitochondrial content (*n* = 3). **i**, **n** Annexin V–FITC and PI detection of apoptosis (*n* = 3). **j**, **o** Detection of β-gal activity by β-gal staining (*n* = 3). **k**, **l** qPCR analysis of mtDNA (*n* = 3). In (**d**), A = BMSCs, B = BMSCs/EGFP, C = BMSCs/shP53, D = BMSCs/Parkin, E = BMSCs/shP53/Parkin. In (**b**), (**c**), (**e**), (**f**), and (**k**–**o**), data are presented as means ± SD. Statistical significances were calculated by ANOVA. In (**b**, **c**) and (**e**, **f**), data were compared with the BMSCs and BMSCs/EGFP groups: *P < 0.05. In (**k**–**o**), data were compared with the BMSCs and BMSCs/EGFP groups: *P < 0.05; or with the BMSCs/shP53 and BMSCs/Parkin groups: ^#^*P* < 0.05. shP53 = protein 53 short-hairpin ribonucleic acid.

When we further downregulated P53 expression via the short-hairpin ribonucleic acid (shRNA) technique in the Parkin–BMSCs that we had established in the early stage, qPCR and western blot results confirmed that the BMSCs overexpressed Parkin, while P53 expression was significantly downregulated (Fig. 5a–f). We also treated BMSCs with 1000 μM H_2_O_2_ for 24 h, which caused them to suffer from OS. The results showed that upregulation of Parkin and downregulation of P53 could further increase the number of mitophagic bodies in BMSCs on the basis of Parkin upregulation alone (Fig. 5g), and MitoTracker Green staining showed that damaged-mitochondria content in the cells also decreased (Fig. 5h, k–m). We further evaluated stress-induced apoptosis and senescence of BMSCs by quantitative detection of Annexin V–FITC/PI, β-gal staining, and aging-related proteins. The results showed that under OS, Parkin upregulation and P53 downregulation in BMSCs significantly decreased the apoptosis rate (Fig. 5i, n), and senescence phenotypes such as β-gal activity also decreased significantly (Fig. 5j, o). This evidence suggested that upregulating Parkin and downregulating P53 expression could significantly enhance mitophagy, thereby removing damaged mitochondria from cells and enabling BMSCs to effectively resist stress apoptosis and aging. It also showed that under OS, P53 also participated in regulation of mitophagy.

### Mechanism of P53 regulating mitophagy

**Fig. 6:**
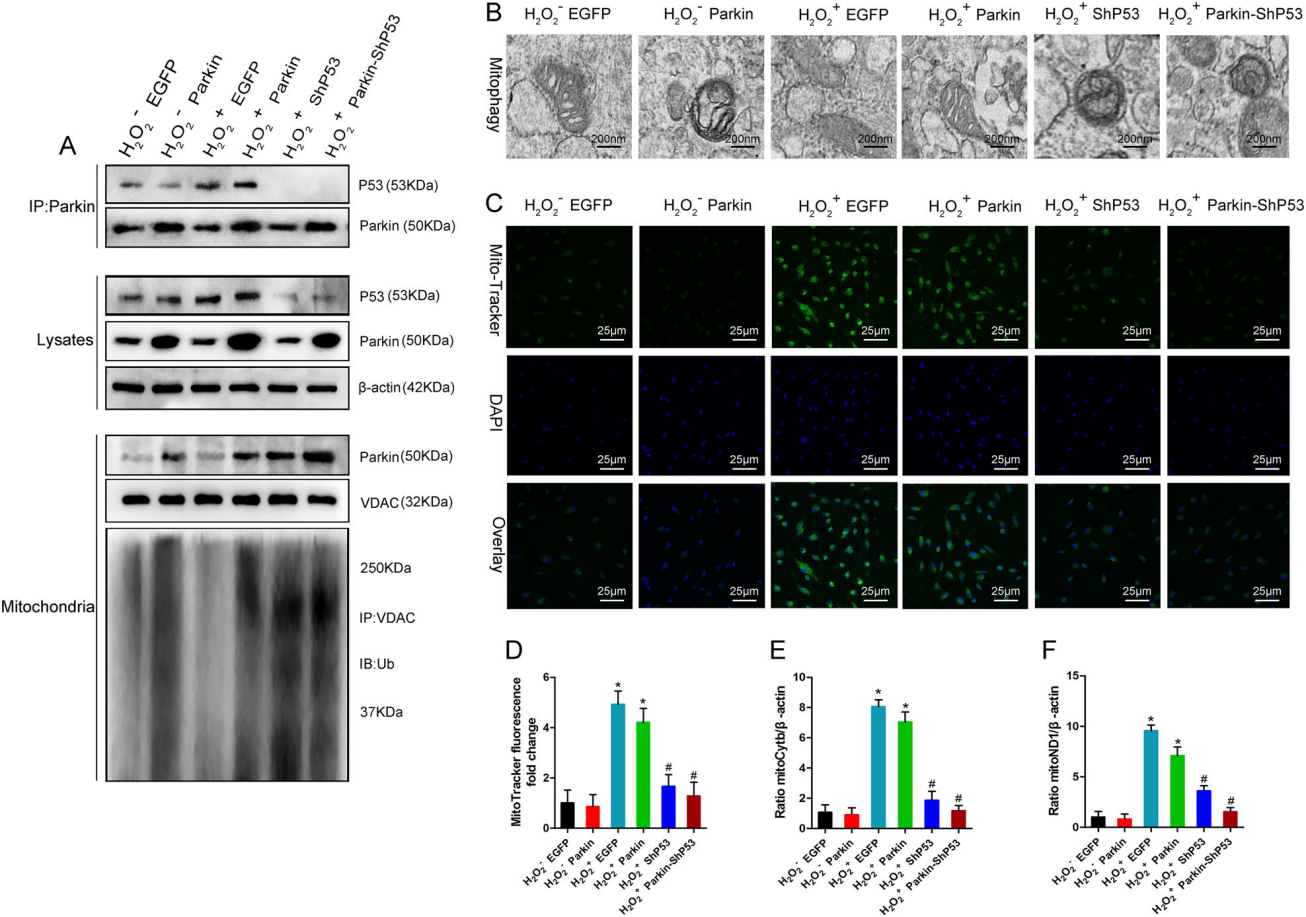
P53 regulated mitophagy via the PINK1–Parkin pathway. **a** P53 interacted with Parkin to inhibit Parkin mitochondrial translocation and mitochondrial-membrane protein ubiquitination (*n* = 3). **b** Observation of mitophagy by TEM (*n* = 3). **c**, **d** MitoTracker Green analysis of mitochondrial content (*n* = 3). **e**, **f** qPCR analysis of mtDNA (*n* = 3). In (**d**–**f**), data are presented as means ± SD. Statistical significances were calculated by ANOVA. Data were compared with the H_2_O_2_^−^ EGFP and H_2_O_2_^−^ Parkin groups: *P < 0.05. Data were compared with the H_2_O_2_^+^ EGFP and H_2_O_2_^+^ Parkin groups: ^#^*P* < 0.05. PINK1 = phosphatase and tensin homolog (PTEN)-induced putative kinase protein 1.

OS can upregulate the expression of P53 in cells^40,41^. Some studies have shown that P53 can interact with Parkin’s RING0 region^30,31^. The PINK1–Parkin pathway is the most important pathway by which mitophagy is mediated^42^; therefore, to further study whether P53 regulated mitophagy via this pathway, we continued to treat BMSCs for 24 h using an H_2_O_2_ (1000 μM)-simulated OS. The results of qPCR and western blot confirmed that P53 expression was upregulated in the H_2_O_2_^+^ EGFP and H_2_O_2_^+^ Parkin groups, compared with the H_2_O_2_^−^ EGFP and H_2_O_2_^−^ Parkin groups. Subsequently, our immunocoprecipitation and immunoblot results showed that P53 upregulation increased the interaction between P53 and Parkin, while decreasing mitochondrial translocation of Parkin and the ubiquitination level of VDAC1 (Fig. 6a). Transmission electron microscopy (TEM) showed that the number of mitophagic bodies decreased (Fig. 6b); MitoTracker Green staining and mtDNA quantification showed that damaged-mitochondria content in cells was significantly increased (Fig. 6c–f). After we downregulated P53 expression of in BMSCs using shRNA, the interaction between P53 and Parkin in the H_2_O_2_^+^ shP53 and H_2_O_2_^+^ Parkin/shP53 groups was reduced, compared with the H_2_O_2_^+^ EGFP and H_2_O_2_^+^ Parkin groups. In addition, mitochondrial translocation of Parkin and the ubiquitin level of VDAC1 increased, the level of mitophagy increased, and the content of damaged mitochondria in cells was significantly decreased (Fig. 6a–f). At the same time, we could see that upregulation of Parkin and downregulation of P53 significantly increased Parkin mitochondrial translocation and ubiquitination of mitochondrial extracorporeal-membrane proteins, as well as significantly enhancing mitophagy (Fig. 6a–f). These results suggested that P53 could regulate mitophagy via the PINK1–Parkin pathway.

### Parkin–shP53 BMSCs repaired early steroid-induced ONFH

**Fig. 7:**
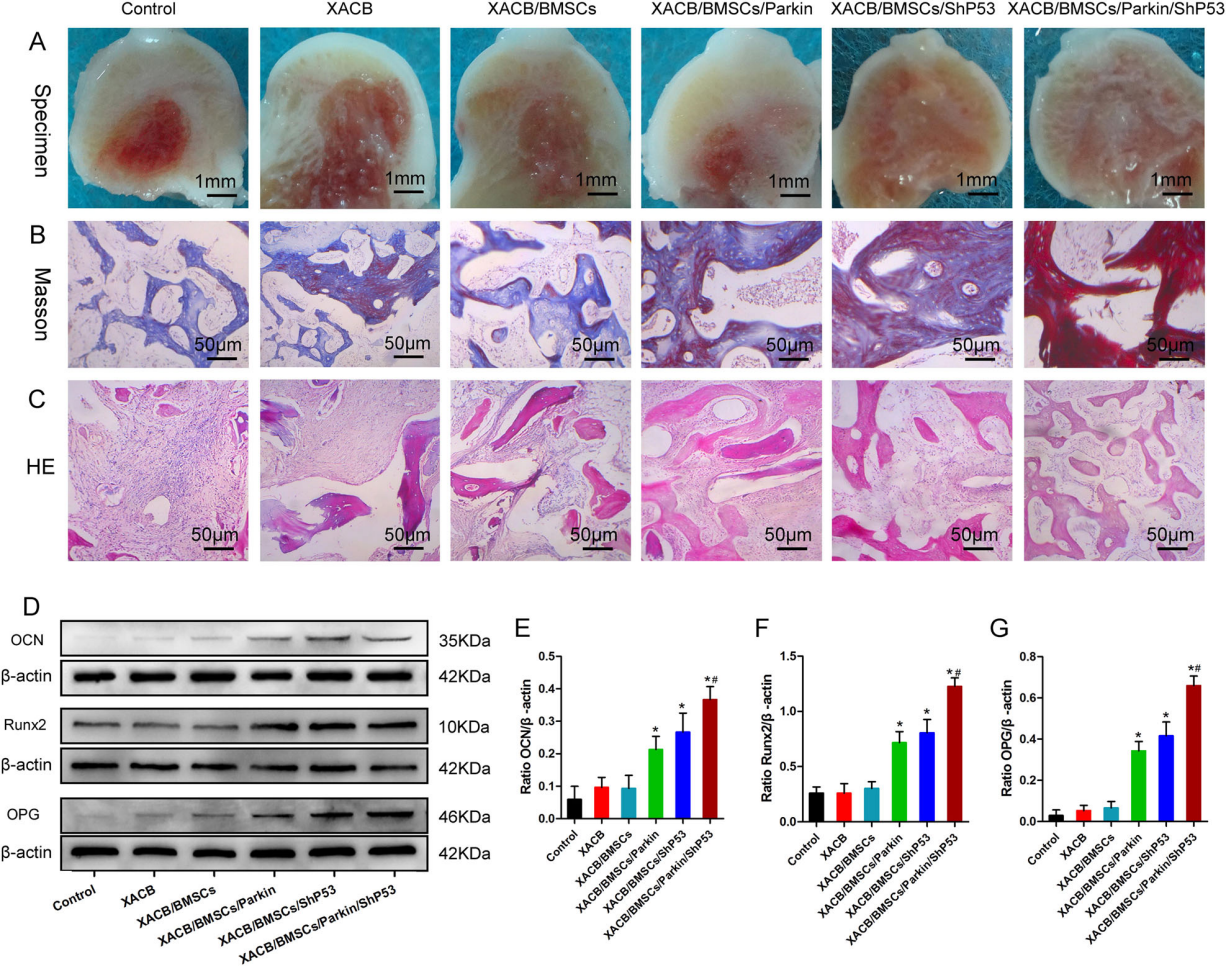
P53 and Parkin co-regulated mitophagy in BMSCs to promote the repair of early steroid-induced ONFH. **a** Gross specimens in which we observed repair of osteonecrosis (*n* = 3). **b** Masson staining for evaluating new-bone maturation (*n* = 3). **c** H&E staining for evaluating new-bone formation (*n* = 3). **d**–**g** Western blot analysis of OCN, Runx2, and OPG expression (*n* = 3). Control was treated with lesion debridement (LD) without transplantation. In (**e**–**g**), data are presented as means ± SD. Statistical significances were calculated by ANOVA. Data were compared with the control, XACB, and XACB/BMSCs groups: *P < 0.05. Data were compared with the XACB/BMSCs/Parkin and XACB/BMSCs/shP53 groups: ^#^*P* < 0.05.

In the OS microenvironment that forms in the avascular necrotic area of the femoral head, accumulation of damaged mitochondria causes apoptosis and senescence of transplanted BMSCs, which seriously affects the transplantation effect. Our results showed that upregulation of Parkin expression and downregulation of P53 expression could significantly enhance mitophagy and then clear damaged mitochondria from cells, which could help BMSCs effectively resist stress apoptosis and aging (Fig. 5). To further evaluate whether enhanced mitophagy could further improve the reparative effect of BMSCs on early steroid-induced ONFH, we first co-transfected BMSCs with Lv-Parkin and Lv-shP53 so that the BMSCs would upregulate Parkin and downregulate P53 simultaneously. Then, we co-cultured these BMSCs with XACB to construct tissue-engineered bone, which we used to repair early steroid-induced ONFH model in rabbits. At 12 weeks postsurgery, observation of gross specimens showed that the defect area in the XACB/BMSCs/Parkin and XACB/BMSCs/shP53 groups were significantly smaller than in the control, XACB, and XACB/BMSCs groups, but in all five of these groups the defect area was not completely repaired. However, in the XACB/BMSCs/Parkin/shP53 group, the area was completely repaired (Fig. 7a).

Next, we evaluated new-bone formation by H&E staining (Fig. 7c) and new-bone maturation by Masson staining (Fig. 7b). Total protein was extracted from femoral-head tissue, and levels of osteogenic markers such as Runx2, OCN, and OPG were detected by western blot (Fig. 7d–g). The results showed that new-bone formation and osteogenic-marker levels in the XACB/BMSCs/Parkin, XACB/BMSCs/shP53, and XACB/BMSCs/Parkin/shP53 groups were higher than in the control, XACB, and XACB/BMSCs groups. Compared with the other groups, however, both metrics were highest in the XACB/BMSCs/Parkin/shP53 group, the new-bone tissue had completely matured, and the boundary between new and normal bone was not clear (Fig. 7b–g). These results suggested that upregulation of Parkin and downregulation of P53 to enhance mitophagy of BMSCs could effectively improve the reparative effect of BMSCs on early steroid-induced ONFH.

## Discussion

BMSCs are used to treat early steroid-induced ONFH^43–45^; their survival and stemness in the bone necrotic area are key to the effectiveness of transplantation^46,47^. Studies have revealed an OS microenvironment in the femoral-head necrotic area in which transplanted BMSCs incur a great deal of stress apoptosis and aging, which seriously limits transplantation effectiveness^11–13^. More and more studies have shown that mitochondrial dysfunction and damaged-mitochondria accumulation occur before cell stress apoptosis and senescence^19–22^. In this study, we preliminarily confirmed that the accumulation of damaged mitochondria was an important cause of BMSCs stress apoptosis and aging. Clearance of these mitochondria mainly depends on mitophagy mediated by the PINK1–Parkin pathway^48^. Our further experiments confirmed that under OS, the decrease in mitophagy mediated by this pathway could lead to accumulation of damaged mitochondria. OS led to upregulation of P53 expression; P53 interacted with Parkin to inhibit Parkin’s mitochondrial translocation and further activation of E3 ubiquitin ligase, which in turn reduced VDAC1 ubiquitination and mitophagy levels. Clearance of damaged mitochondria was obstructed, which eventually led to their accumulating in cells. By using the shRNA technique to downregulate P53 expression, we could effectively reverse the above process. However, in this study we did not further investigate the mechanism of P53 upregulation induced by OS or the interaction site between P53 and Parkin. Related studies in cardiomyocytes and neurons indicate that OS increases P53 expression mainly by initiating the DNA damage response (DDR) and mammalian target of rapamycin (mTOR) pathways^26,27^. After its expression is upregulated, P53 can bind to the RING0 region of Parkin and then interfere with Parkin’s biological function^30,31^. Whether this is also true in BMSCs requires further study.

Under OS, mitochondria suffer functional damage and accumulate in cells, releasing excessive ROS and apoptosis-inducing factors, which not only oxidatively damage DNA, protein, lipid, and other biological macromolecules, but also activate c-Jun N-terminal kinase (JNK), P38MAPK, DDR, and P53 pathways, ultimately leading to cell stress apoptosis and senescence^21,22,49–51^. Therefore, accumulation of damaged mitochondria is an important cause of stress apoptosis and aging in BMSCs^52^. When we further downregulated P53 expression in the BMSCs/Parkin group, mitochondrial translocation of Parkin and mitophagy were increased, significantly reducing damaged mitochondria and ROS in the cells; this was effective against BMSCs stress-induced apoptosis and senescence. Subsequent in vivo studies have also confirmed that upregulating Parkin while downregulating P53 to enhance mitophagy can improve the reparative effect of BMSCs on early steroid-induced ONFH. However, due to limitations imposed by experimental conditions, we could not directly observe survival and differentiation of BMSCs in the osteonecrotic area in vivo, which needs further confirmation by subsequent experiments.

**Fig. 8:**
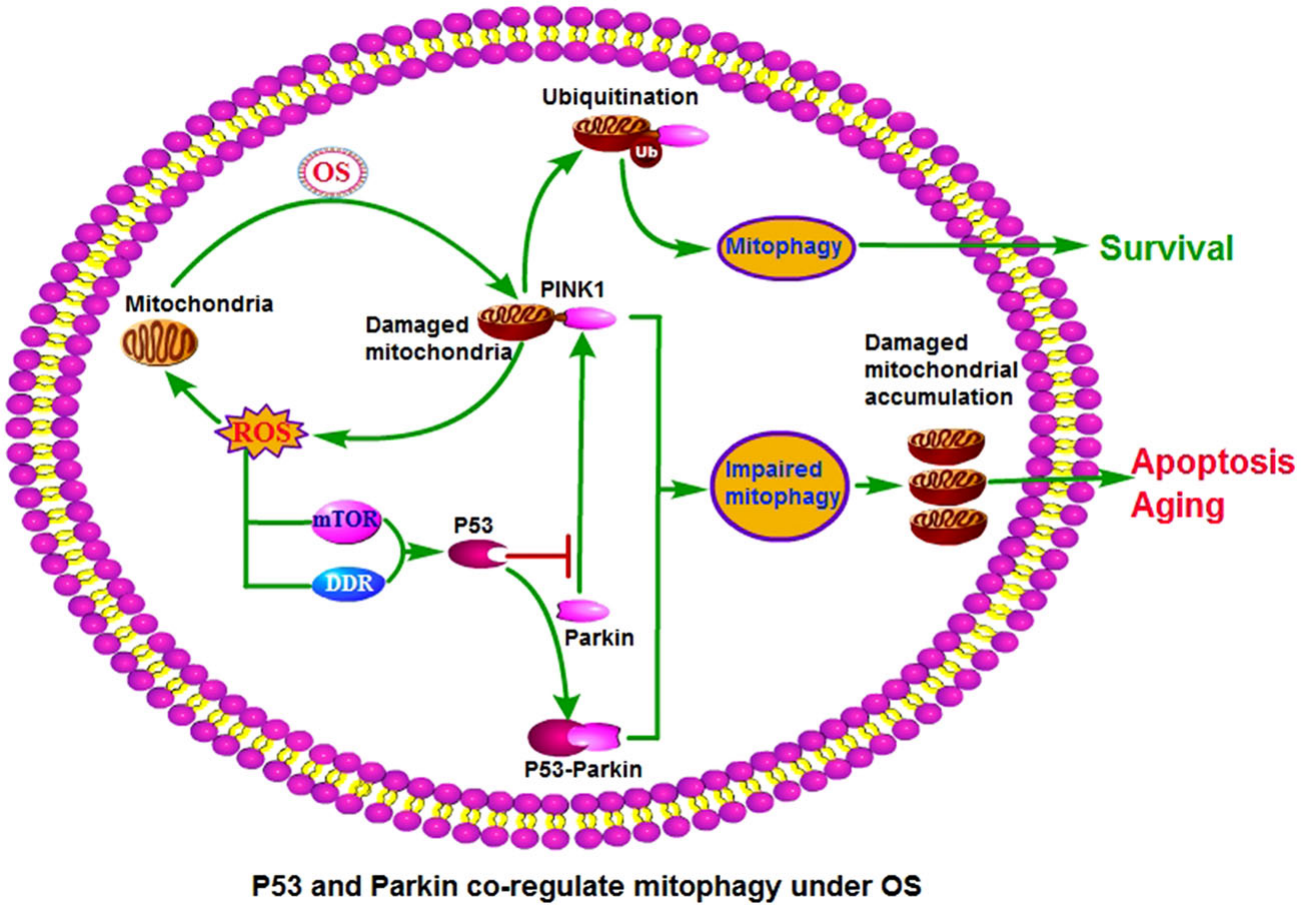
Hypothetical model of P53 and Parkin co-regulating mitophagy in BMSCs to interfere with cell stress-induced apoptosis and senescence. Under OS, mitochondrial function is impaired and damaged mitochondria release excessive ROS, which can further damage the mitochondria, creating a vicious circle. Moreover, ROS can upregulate P53 expression via the DDR and mTOR pathways. P53 binds to Parkin and inhibits its mitochondrial translocation, the ubiquitination of mitochondrial outer-membrane proteins was inhibited. Eventually, the level of mitophagy decreased, and a large number of mitochondria accumulated in the cells, resulting in apoptosis and senescence of the cells. DDR = DNA damage response; mTOR = mammalian target of rapamycin.

In conclusion, despite the limitations and other deficiencies of this study, we preliminarily confirmed the role and mechanism of P53 and Parkin in regulating mitophagy in BMSCs (Fig. 8). We also proved that upregulating Parkin and downregulating P53 could significantly enhance mitophagy in BMSCs, resist BMSCs stress-induced apoptosis and senescence, and effectively improve the reparative effect of BMSCs on early steroid-induced ONFH.

## Methods

### Animals

All animal studies were approved by the Experimental Animal Bioethics Committee of Guizhou Medical University, Guiyang, China. All animal procedures conformed to the *Guide for the Care and Use of Laboratory Animals* published by the Directive 2010/63/EU of the European Parliament. We extracted BMSCs from 20 young male New Zealand white rabbits (4–6 weeks old; 1.0–2.0 kg) and built ONFH models using 180 adult male New Zealand white rabbits (4.0–5.0 kg). All rabbits were provided by the Laboratory Animal Center of Guizhou Medical University.

### Cell culture

Our methods for cultivating BMSCs were similar to those in our previous studies^53^. From the young male rabbits, we extracted bilateral distal-femur and proximal-tibia bone marrow under sterile conditions and added an appropriate amount of heparin/phosphate-buffered saline (PBS; 1000 U/mL) to prevent blood coagulation. We then diluted the bone marrow solution 1:1 with PBS buffer (HyClone, Illinois, USA), blew it into a single-cell suspension, centrifuged it at 600 g/min for 5 min, and removed the upper layer of suspended fat. Then the precipitated bone marrow was slowly added to Percoll solution (Pharmacia, New York, USA) dropwise along the wall of the tube to a density of 1.073 g/mL and centrifuged at 900 g/min for 30 min. We harvested nucleated cells, washed them in PBS buffer, and centrifuged them at 600 g/min for 15 min. The cells were resuspended in complete L-glutamine Dulbecco’s modified Eagle medium (L-DMEM; GIBCO, Massachusetts, USA) containing 10% fetal bovine serum (FBS; GIBCO) and 1% double antibody (HyClone), and cultured at 37 °C under 5% CO_2_. When primary BMSCs had pooled at 80–90% of the bottom of the flask, we digested the cells with an appropriate amount of 0.25% trypsin–0.02% ethylenediaminetetraacetic acid (EDTA; GIBCO) at 37 °C and passaged them at 1:3. Third-generation BMSCs were used for subsequent experiments.

### Osteogenesis induction and identification of BMSCs

We induced osteogenesis in the BMSCs using the same method as in our previous studies^3^. Cell density of third-generation BMSCs was adjusted to 1 × 10^5^/mL, and the cells were inoculated on a 6-well plate with 2 mL per well. When cell confluence reached 60–70%, we replaced the experimental group with osteogenic-differentiation medium (Cyagen Biosciences, Guangzhou, China) but left the control group in complete L-DMEM. After osteogenic induction for 2 weeks, PBS buffer was sucked out, cells were fixed, calcium nodules were identified by 0.1% alizarin red staining (Cyagen Biosciences), and ALP activity was detected by a modified Gomori calcium cobalt method (Solarbio, Beijing, China).

### Lentivirus infection

We purchased Lv-P53-EGFP, Lv-shP53-EGFP, and Lv-Parkin-EGFP from Shanghai GeneChem Co., Ltd. (Shanghai, China) A pre-transfection experiment revealed the best multiplicity of infection (MOI; 100) and best transfection conditions (enhanced infection solution ([Eni.s] + Polybrane; both from Shanghai GeneChem). We infected BMSCs with lentivirus and replaced the infected medium with complete L-DMEM 10 h later. On day 4 after infection, was added L-DMEM containing 2 μg/mL puromycin (Shanghai GeneChem) to screen stable strains. When all cells in the blank control had died, we reduced the concentration of puromycin to 1 μg/mL to maintain screening.

### Real-time PCR (RT-PCR)

We extracted RNA from BMSCs via column affinity purification (QIAGEN, Hilden, Germany) and synthesized complementary DNAs (cDNAs) using M-MuLV RT Master Mix with Oligo(dT) (Sangon Biotech, Shanghai, China). We performed RT-PCR on a StepOnePlus system (Applied Biosystems, California, USA) in 96-well plates with specific primers and SYBR Green Mix (Sangon Biotech). Rabbit primers (Sangon Biotech) were as follows: Parkin-F: TGACCAGTTGCGTGTGATCTTCG; Parkin-R: GTTGTCTCCTCCAGGCGTGTTG; P53-F: ATGGAGGAG TCGCAGTCGGATC; P53-R: GGTGGTCAGCAGGTTGTTCTCAG; ACTB-F: TCCCTGGAGAAGAGCTACGA; ACTB-R: GTACAGGT CCTTGCGGATGT. We calculated the fold change value of mRNA expression over that of control using the ^ΔΔCt^ method.

### Isolation of mitochondria and cytoplasm, and extraction of protein

We isolated mitochondrial and cytoplasmic fractions using a commercial kit (QIAGEN). For whole-cell lysates, cells were lysed with radioimmunoprecipitation assay (RIPA) cell lysate (Beyotime Institute of Biotechnology, Shanghai, China) and centrifuged at 13,000 g/min for 10 min to obtain supernatant. All work was performed on wet ice. We quantified proteins using a bicinchoninic acid (BCA) assay (Solarbio) and stored them at −80 °C.

### Immunoprecipitation and immunoblot

We incubated the cytosolic fractions with antibodies for 12 h at 4 °C and then added Protein A/G Plus-Agarose (Sigma-Aldrich, Darmstadt, Germany) for 3 h at 4 °C on a rotating device. Immunoprecipitates were collected by centrifugation at 6000 g/min at 4 °C and washed with lysis buffer (20 mM Tris pH 7.5, 150 mM NaCl, 1 mM EDTA, 1% Triton X-100, proteases, and phosphatase inhibitors; Sigma-Aldrich). We eluted the pellets by heating them at 95 °C for 5 min in electrophoresis sample buffer and subjected them to immunoblotting. For immunoblotting, we prepared sodium dodecyl sulfate polyacrylamide gel electrophoresis (SDS-PAGE) gel (Solarbio), added an equal amount of protein for electrophoresis, and transferred the mixture to a polyvinylidene fluoride (PVDF) membrane (Merck Millipore, Darmstadt, Germany). We used rabbit anti-P53 (1:1000; ab154036; Abcam, Cambridge, UK), rabbit anti-Parkin (1:300; BA1682-1; Boster Biological Technology, Ltd., Wuhan, China), rabbit anti-VDAC1 (1:1000; ab34726; Abcam), mouse anti-ubiquitin (1:1000; ab52664; Abcam), rabbit anti-NDUFA9 (1:1000; ab128744; Abcam), mouse anti-mitochondrial 70 kDa heat shock protein (mtHsp70; 1:2000; H5147; Sigma-Aldrich), rabbit anti-P21 (1:1000; bs-10129R; Bioss, Shanghai, China), rabbit anti-P16 (1:1000; A00016-3; Boster), rabbit anti-OPG (1:1500; bs-20624R; Bioss), mouse anti-OCN (1:1000; ab13420; Abcam), rabbit anti-Runx2 (1:1000; AV36678; Sigma-Aldrich), and rabbit anti-β-actin (1:3000; ab8227; Abcam) for primary-antibody response; and horseradish peroxide (HRP)-conjugated goat anti-rabbit and goat anti-mouse immunoglobulin G (IgG; Beyotime) for secondary-antibody response. We used electrochemiluminescence (ECL; Merck Millipore) used for exposure, took photographs using a gel imaging system (Clinx Science Instruments, Ltd., Shanghai, China), and performed quantitative analysis using ImageJ software (1.4.3.67).

### Cellular oxidative stress

In accordance with our previous research^35^, we created a classic OS BMSCs model by treating BMSCs with a high concentration of H_2_O_2_ (1000 μM) for 24 h. L-DMEM was used as the control. After P53 knockout and Parkin overexpression in BMSCs per experimental-grouping conditions, we treated the BMSCs in each group with H_2_O_2_ (Chengdu Jinshan Chemical Reagent Co., Ltd., Sichuan, China) for 24 h and subjected them to OS.

### DCFH-DA staining

After we washed the cells three times in PBS buffer, we prepared the reaction mixture reagent using a 2′,7′-Dichlorodihydrofluorescein diacetate (DCFH-DA) fluorescent-probe kit (Sigma-Aldrich) per manufacturer’s instructions. Cells were incubated at 37 °C for 30 min. We observed green fluorescence under a confocal microscope (Zeiss, Oberkochen, Germany) in the fluorescein isothiocyanate (FITC) green-fluorescence channel.

### JC-1 staining

We washed the cells three times in PBS buffer and prepared a JC-1 fluorescent probe using a MMP detection kit (Nanjiang KeyGen Biotech Co., Ltd, Jiangsu, China) per manufacturer’s instructions. After staining at 37 °C for 30 min, we observed red and green fluorescence under a confocal microscope (Zeiss).

### MitoTracker Green staining

We washed the cells three times in PBS buffer and prepared MitoTracker Green stock solution and working solution using a MitoTracker Green fluorescent probe kit (Beyotime) per manufacturer’s instructions. Cells were incubated with the working solution at 37 °C for 30 min. We observed green fluorescence in the FITC green-fluorescence channel using a laser confocal microscope (Zeiss).

### β-gal staining

We fixed the cells at room temperature (RT) for 15 min and prepared a staining working solution using a cell senescence β-gal staining kit (Beyotime) per manufacturer’s instructions. After the working solution was added, the cells were incubated at 37 °C overnight and observed using a general optical microscope.

### TUNEL–DAPI staining

We fixed the cells in 4% paraformaldehyde for 30 min, permeabilized them with 0.3% Triton X-100 for 5 min at RT, and prepared a TUNEL assay solution using a TUNEL Apoptosis Detection Kit (Beyotime) per manufacturer’s instructions. The assay solution and cells were incubated at 37 °C for 60 min in the dark and stained with 20 μg/mL DAPI (Solarbio) for 3 min. We then observed red and blue fluorescence using a laser confocal microscope (Zeiss).

### Annexin V–FITC/PI detection of apoptosis

Using an Annexin V**–**FITC Apoptosis Detection Kit (BD Biosciences, San Jose, California, USA) per manufacturer’s instructions, we directly added 5 μL Annexin V**–**FITC and 5 μL PI, gently vortexed the cells, and incubated them at RT for 15 min in the dark. We then detected apoptosis in the cells via flow cytometry (Beckman Coulter, Indianapolis, Indiana, USA).

### TEM observation of mitophagy

We trypsinized the cells, collected the cell pellet, added 2.5% glutaraldehyde solution, and fixed the cells at 4 °C overnight. After dehydration, embedding, ultra-thin sectioning, uranium dyeing, and lead staining, we observed mitophagy using a TEM (Hitachi, Tokyo, Japan).

### XACB

We removed cortical bone and cartilage from fresh porcine vertebrae (Laboratory Animal Center of Guizhou Medical University, Guizhou, China) according to the procedures established by Li et al. and Long et al.^54–56^. Cancellous-bone blocks were shaped into cylinders 6 mm in diameter at the bottom and 20 mm high using a cutting and molding device. After repeated cleanings with distilled water, we subjected the cylinders to 30% H_2_O_2_ deproteinization for 48 h, 1:1 chloroform/methanol degreasing for 24 h, freeze-drying in a −50 °C freeze dryer for 24 h, ethylene oxide closed disinfection for 24 h to obtain sterile XACB.

### Detection of biocompatibility between BMSCs and XACB

Based on our previous research^3^, we cut XACB into small cylinders 5 mm in diameter at the bottom and 5 mm high and immersed them in complete L-DMEM for 24 h. We added BMSCs suspension (1 × 10^7^/mL) to XACB, allowed the cells to adhere for 3 h in an incubator (37 °C, 5% CO_2_), slowly added complete L-DMEM along the wall of the culture plate, and continued to culture the mixture in the incubator (37 °C, 5% CO_2_) to construct tissue-engineered bone (BMSCs/XACB). Each day for the next week, we took five pieces of BMSCs/XACB out of the incubator, collected cells from them via digestion and centrifugation, and added 10 μL of Cell Counting Kit-8 (CCK-8) solution (Solarbio) to each well. We measured the absorbance value (450 nm) until day 7, when we drew the growth curve based on culture time and absorbance value. We cultured XACB and BMSCs on day 6 and observed the growth of BMSCs on the surface of XACB using a SEM (Hitachi).

### Early steroid-induced ONFH rabbit model

After weighing and disinfection, we injected LPS (10 μg/kg/d; Sigma-Aldrich) into the ear veins of 180 adult male rabbits for 2 days. On day 2, after LPS injection, we injected methylprednisolone (40 mg/kg/d; Pfizer) into the rabbits’ gluteal muscles for 3 days, weighed before each injection^57–60^. Rabbits in the control group were injected with saline. Eight weeks after modeling, we stained femoral-head tissue with H&E, and the successful model was used for in vivo experiments.

### Transplantation of tissue-engineered bone

Rabbits were shaven and their skin cleaned 1 week before surgery. We injected penicillin (50,000 U/kg; CSPC Pharmaceutical Group, Ltd, Hebei, China) intramuscularly 30 min before surgery to prevent infection. We anesthetized the rabbits with 3% pentobarbital (1 mL/kg; Merck Millipore) by ear vein, put them into a prone position, and disinfected the surgical site. A posterior lateral-arc incision of about 5 cm was made to the right hip joint; after exposing this joint, we exposed the femoral head using a T-shaped incision. A 3-mm diameter spherical grinding drill was used at the junction of bone and cartilage, drilling from the posterolateral side to the anteromedial side of the femoral head at a depth of 4–6 mm (the diameter of the femoral head in adult rabbits is 6–8 mm); and then lesion debridement (LD) was performed with a curette. We implanted BMSCs/XACB according to experimental grouping: control (LD), XACB, XACB/BMSCs, XACB/BMSCs/Parkin, XACB/BMSCs/shP53, or XACB/BMSCs/Parkin/shP53. A gelatin sponge (Baotou Dongbao Biotechnology Co., Ltd, Shenzhen, China) was then inserted into the tunnel. Operations were performed on the right side, and the left side was the self-control. Penicillin (50,000 U/kg) was used continuously for 3 days after the operation to prevent infection. Randomization was used to determine animals were allocated to experimental groups. The operator or investigator was blinded to the grouping.

### X-ray examination

At 12 weeks after surgery, after intravenous anesthesia with 3% pentobarbital sodium (1 mL/kg), we examined all experimental animals by X-ray (60 kV, 50 mA, 0.15 s), with the animals in the prone position.

### Observation of the femoral-head specimen

At 12 weeks after surgery, we removed the femoral head, observed its surface, dissected it longitudinally from the middle, and observed repair of the femoral-head defect.

### H&E and Masson staining

At 12 weeks after surgery, bone tissue was routinely decalcified, dehydrated, and paraffin-embedded to make 3-μm-thick sections, which we stained using a H&E staining kit (Solarbio) per manufacturer’s instructions. Paraffin sections were dewaxed to water and stained, made transparent, mounted, and microscopically examined using a Masson’s trichrome staining kit (Solarbio) per manufacturer’s instructions.

### Extraction of total protein from bone tissue

We took out the femoral head and removed the normal bone and cartilage tissue on its surface with a rongeur; the remaining bone tissue was washed with PBS. We crushed the bone tissue using a hammer tissue pulverizer and then quickly transferred the fragments into a mortar containing liquid nitrogen. The bone tissue was ground into a powder (with adequate liquid nitrogen maintained during grinding), the powdered bone tissue was collected into an Eppendorf (EP) tube, and a protein lysate was added to each tube (200 μL lysate per 100 mg bone tissue). After cleavage on ice for 30 min, we pipetted the lysate from the EP tube into a second EP tube, centrifuged it at 12,000 rpm for 15 min at 4 °C, and harvested the supernatant for western blot analysis.

### Statistical analysis

All statistical data were calculated and graphed using GraphPad Prism software version 6 (GraphPad Software, San Diego, California, USA). To assess statistical significance, we used a Kolmogorov–Smirnov test to analyze normal distribution of data, a two-tailed unpaired Student’s *t* test for analyses involving only two groups for comparison, and analysis of variance (ANOVA) and appropriate post hoc tests for analyses involving >2 groups for comparisons. All error bars are expressed as mean ± standard deviation (SD). *P* < 0.05 was considered statistically significant.

## Supplementary information

Supplementary figure 1

Supplementary figure legends

## Data Availability

The additional data or reagents are available from the corresponding author upon reasonable request.
