# Supplementary figures and images for: P53 and Parkin co-regulate mitophagy in bone marrow mesenchymal stem cells to promote the repair of early steroid-induced osteonecrosis of the femoral head
Source: Cell Death Dis. 2020 Jan 20;11(1):42. doi: 10.1038/s41419-020-2238-1 (PMC6971291; doi:10.1038/s41419-020-2238-1)

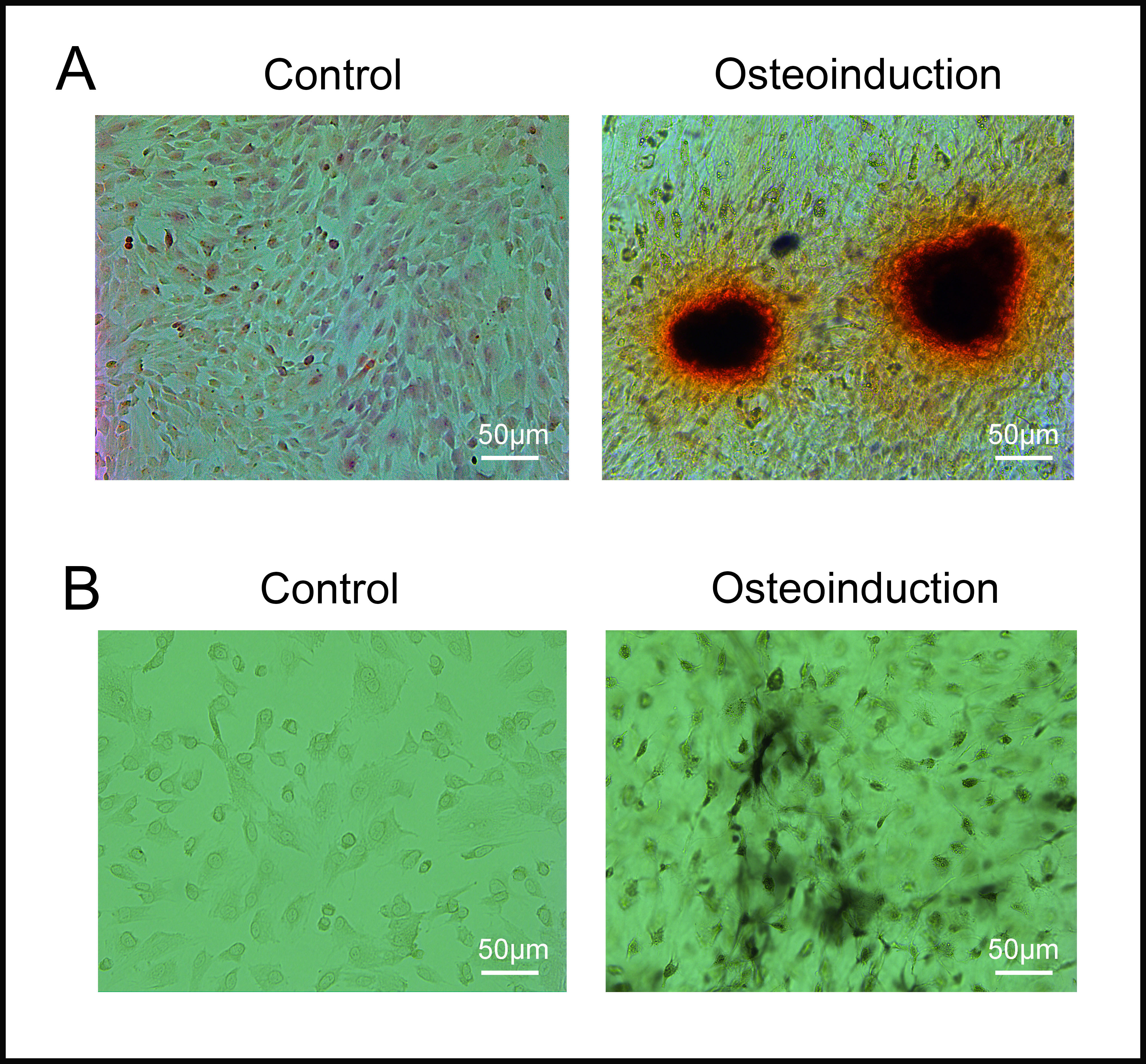

Supplement: Supplementary file 1 — Supplementary figure 1 [file 41419_2020_2238_MOESM1_ESM.jpg]
