# Supplementary material for: P53 and Parkin co-regulate mitophagy in bone marrow mesenchymal stem cells to promote the repair of early steroid-induced osteonecrosis of the femoral head
Source: Cell Death Dis. 2020 Jan 20;11(1):42. doi: 10.1038/s41419-020-2238-1 (PMC6971291; doi:10.1038/s41419-020-2238-1)
Supplement: Supplementary file 2 — Supplementary figure legends [file 41419_2020_2238_MOESM2_ESM.pdf]

Supplementary figure legends

Figure S1. Osteogenesis of BMSCs in vitro. **A** Alizarin red staining after induction of BMSCs osteogenesis. **B** Alkaline phosphatase (ALP) staining after induction of BMSCs osteogenesis. Osteogenic-induction medium was used for osteogenic differentiation, and L-glutamine Dulbecco's Modified Eagle Medium (L-MDEM) was used as control.
